# Recombination Every Day: Abundant Recombination in a Virus during a Single Multi-Cellular Host Infection

**DOI:** 10.1371/journal.pbio.0030089

**Published:** 2005-03-01

**Authors:** Remy Froissart, Denis Roze, Marilyne Uzest, Lionel Galibert, Stephane Blanc, Yannis Michalakis

**Affiliations:** **1**Biologie et Génétique des Interactions Plante-Parasite, Unité Mixte de Recherche Centre de Coopération Internationale en Recherche Agronomique pour le Développement (CIRAD)–Institut National de la Recherche Agronomique (INRA)–Ecole National Supérieure Agronomique de Montpellier (ENSAM)TA 41/K, Campus International de Baillarguet, MontpellierFrance; **2**Génétique et Evolution des Maladies Infectieuses, Unité Mixte de Recherche Centre National de la Recherche Scientifique (CNRS)–Institut de Recherche pour le Développement (IRD) 2724MontpellierFrance; John Innes CenterUnited Kingdom

## Abstract

Viral recombination can dramatically impact evolution and epidemiology. In viruses, the recombination rate depends on the frequency of genetic exchange between different viral genomes within an infected host cell and on the frequency at which such co-infections occur. While the recombination rate has been recently evaluated in experimentally co-infected cell cultures for several viruses, direct quantification at the most biologically significant level, that of a host infection, is still lacking. This study fills this gap using the cauliflower mosaic virus as a model. We distributed four neutral markers along the viral genome, and co-inoculated host plants with marker-containing and wild-type viruses. The frequency of recombinant genomes was evaluated 21 d post-inoculation. On average, over 50% of viral genomes recovered after a single host infection were recombinants, clearly indicating that recombination is very frequent in this virus. Estimates of the recombination rate show that all regions of the genome are equally affected by this process. Assuming that ten viral replication cycles occurred during our experiment—based on data on the timing of coat protein detection—the per base and replication cycle recombination rate was on the order of 2 × 10^−5^ to 4 × 10^−5^. This first determination of a virus recombination rate during a single multi-cellular host infection indicates that recombination is very frequent in the everyday life of this virus.

## Abstract 2

An analysis of recombination of the cauliflower mosaic virus during an infection reveals that recombination is extremely frequent and provides the first range of estimates for a plant virus

## Introduction

As increasing numbers of full-length viral sequences become available, recombinant or mosaic viruses are being recognized more frequently [1,2,3]. Recombination events have been demonstrated to be associated with viruses expanding their host range [4,5,6,7] or increasing their virulence [8,9], thus accompanying, or perhaps even being at the origin of, major changes during virus adaptation. It remains unclear, however, whether recombination events represent a highly frequent and significant phenomenon in the everyday life of these viruses.

Viruses can exchange genetic material when at least two different viral genomes co-infect the same host cell. Progeny can then become hybrid through different mechanisms, such as reassortment of segments when the parental genomes are fragmented [10], intra-molecular recombination when polymerases switch templates (in RNA viruses) [11], or homologous or non-homologous recombination (in both RNA and DNA viruses). Quantification of viral recombination in multi-cellular organisms has been attempted under two distinct experimental approaches: in vitro (in cell cultures) [12,13,14,15], and in vivo (in live hosts) [16,17,18]. The in vitro approach, which has so far been applied only to animal viruses, allows the establishment of the “intrinsic” recombination rate in experimentally co-infected cells in cell cultures [14,15,19]. However, it does not necessarily reflect the situation in entire, living hosts, where the frequency of co-infected cells is poorly known and depends on many factors such as the size of the pathogen population, the relative frequency and distribution of the different variants, and host defense mechanisms preventing secondary infection of cells. The in vivo experimental approach is closer to biological conditions and may thus be more informative of what actually happens in “the real world.”

However, as discussed below, numerous experimental constraints have so far precluded an actual quantification of the baseline rate of recombination. First, many experimental designs have used extreme positive selection, where only recombinant genomes were viable (e.g., [13,20,21]). Other studies did not use complementation techniques but detected recombinants by PCR within infected hosts or tissues [18,22,23,24,25], which provides information on their presence but not on their frequency in the viral population. So far, no quantitative PCR or other quantitative method has been applied to evaluate the number of recombinants appearing in an experimentally infected live host. Finally, recent methods based on sequence analysis inferred the population recombination rate, rather than the individual recombination rate [1,26,27]. While results from these methods certainly take in vivo recombination into account, there are other caveats: isolates have often been collected in different hosts—sometimes in different geographical regions—and sometimes the selective neutrality of sequence variation on which these estimates are based is not clearly established. Estimates from such studies by essence address the estimation of the recombination rate at a different evolutionary scale.

Taken together, the currently available information indicates that no viral recombination rate has ever been estimated directly at time and space scales corresponding to a single multi-cellular host infection, although this level is most significant for the biology and evolution of viruses. This study intends to fill this gap by evaluating the recombination frequency of the cauliflower mosaic virus (CaMV) during a single passage in one of its host plants (the turnip Brassica rapa).

CaMV is a pararetrovirus, which is a major grouping containing hepadnaviruses (e.g., hepatitis B virus), badnaviruses (e.g., banana streak virus), and caulimoviruses (e.g., CaMV). Pararetroviruses are characterized by a non-segmented double-stranded DNA genome. After entering the host cell nucleus, the viral DNA accumulates as a minichromosome [28] whose transcription is ensured by the host RNA polymerase II [29]. The CaMV genome consists in approximately 8,000 bp and encodes six viral gene products that have been detected *in planta* (Figure 1) [30]. Viral proteins P1 to P6 are expressed from two major transcripts, namely a 19S RNA, encoding P6, and a 35S RNA corresponding to the entire genome and serving as mRNA for proteins P1–P5 [31]. Using the pre-genomic 35S RNA as a matrix, the protein P5 (product of gene V) reverse-transcribes the genome into genomic DNA that is concomitantly encapsidated [30].

**Figure 1:**
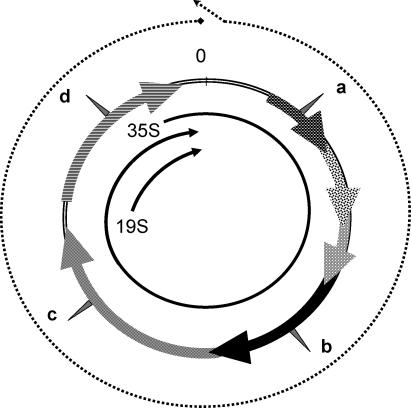
Genetic Map of CaMV The CaMV genome is a circular double-stranded DNA of 8,024 bp, represented in the figure by a double line. The thick arrows with different textures represent the organization of open reading frames I to VI, encoding proteins detected *in planta*. Markers a, b, c, and d were engineered at the positions indicated (see Materials and Methods for precise positions). The inner black arrows represent monocistronic 19S RNA and polycistronic 35S RNA produced by the cellular machinery. The nucleotide position 0 (numbering according to [44]) indicates the origin of replication via reverse transcription, which occurs in the direction indicated by the dotted outermost circle-like arrow. Reverse transcription is accomplished by the viral reverse transcriptase, using the 35S RNA as template [49].

The detection of CaMV recombinants in turnip hosts has been reported numerous times. Some studies have demonstrated the appearance of infectious recombinant viral genomes after inoculation (i) of a host plant with two infectious or non-infectious parental clones [21,32,33,34,35] or (ii) of a transgenic plant containing one CaMV transgene with a CaMV genome missing the corresponding genomic region [36]. While the former revealed inter-genomic viral recombination, the latter demonstrated that CaMV can also recombine with transgenes within the host's genome. Another study based on phylogenetic analyses of various CaMV strains has clearly suggested different origins for different genomic regions and, hence, multiple recombination events during the evolution of this virus [37]. Indirect experimental evidence has indicated that, in some cases, CaMV recombination could occur within the host nucleus, between different viral minichromosomes, presumably through the action of the DNA repair cellular machinery [21,35]. Nevertheless, the mechanism of “template switching” during reverse transcription, predominant in all retroviruses, most certainly also applies to pararetroviruses. For this reason, and on the basis of numerous experimental data, CaMV is generally believed to recombine mostly in the cytoplasm of the host cell, by “legal” template switching between two pre-genomic RNA molecules [21,35,36,38,39], or “illegal” template switching between the 19S and the 35S RNA [36,40]. Under this hypothesis, recombination in CaMV could therefore be considered as operating on a linear template during reverse transcription, with the 5′ and 3′ extremities later ligated to circularize the genomic DNA (position 0 in Figure 1). The above cited studies clearly demonstrate that CaMV is able to recombine. However, since these studies are based on complementation techniques, non-quantitative detection, or phylogenetically based inferences of recombination, they do not inform us on whether recombination is an exceptional event or an “everyday” process shaping the genetic composition of CaMV populations.

In the present work, we aimed at answering this question. To this end, we have constructed a CaMV genome with four genetic markers, demonstrated to be neutral in competition experiments. By co-inoculating host plants with equal amounts of wild-type and marker-containing CaMV particles, we have generated mixed populations in which impressive proportions of recombinants—distributed in several different classes corresponding to exchange of different genomic regions—have been detected and quantified. Altogether, the recombinant genomes averaged over 50% of the population. Further analysis of these data, assuming a number of viral replications during the infection period ranging from five to 20, indicates that the per nucleotide per replication cycle recombination rate of CaMV is of the same order of magnitude, i.e., on the order of a few 10^−5^, across the entire genome. We thereby provide the first quantification, to our knowledge, of the recombination rate in a virus population during a single passage in a single host.

## Results

### Recombinant Frequency in CaMV Populations from Co-Infected Plants

From Figure 1, and supposing that all marker-containing genomic regions can recombine, we could predict the detection and quantification of seven classes of recombinant genotypes: +bcd/a+++, a+cd/+b++, ab+d/++c+, abc+/+++d, ++cd/ab++, a++d/+bc+, and a+c+/+b+d. Indeed, all classes were detected, and their frequencies in the ten CaMV populations analyzed are summarized in Table 1.

**Table 1:**
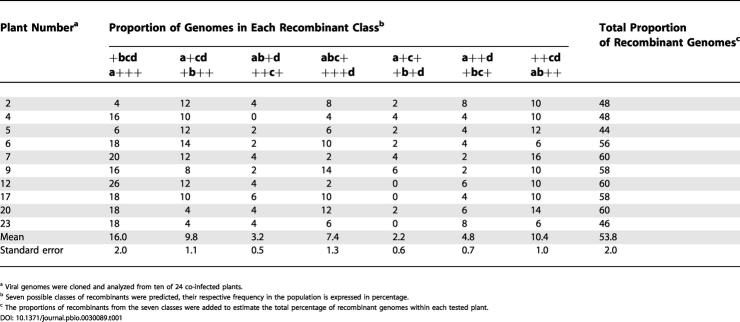
Quantification of Recombinant Genomes in CaMV Populations

^a^ Viral genomes were cloned and analyzed from ten of 24 co-infected plants

^b^ Seven possible classes of recombinants were predicted, their respective frequency in the population is expressed in percentage

^c^ The proportions of recombinants from the seven classes were added to estimate the total percentage of recombinant genomes within each tested plant

Altogether, the proportion of recombinant genomes found in the mixed viral populations was astonishingly high and very similar in the ten co-infected plants analyzed (Table 1, last column), ranging between 44% (plant 5) to 60% (plants 7, 12, and 20), with a mean frequency (± standard error) of 53.8% ± 2.0%. This result indicates that recombination events are very frequent during the invasion of the host plant by CaMV and represents, to our knowledge, the first direct quantification of viral recombination during the infection of a live multi-cellular host.

### Probability of Recombination between Various Pairs of Markers

The inferred per generation recombination and interference rates, assuming that CaMV undergoes ten replication cycles during the 21 d between infection and sampling, are given for each of the ten plants in Table 2. Recombination rates between adjacent markers are large, on the order of 0.05 to 0.1. Taking the distance in nucleotides between markers into account yields an average recombination rate per nucleotide and generation on the order of 4 × 10^−5^. Interestingly, this recombination rate does not vary throughout the genome (Kruskal–Wallis test, *p* = 0.16).

**Table 2:**
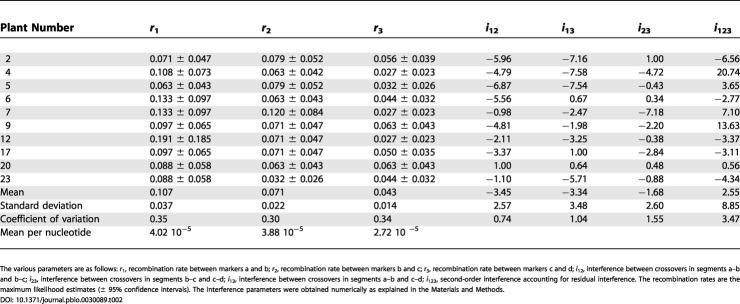
Recombination Parameters for the Viral Populations Sampled from Ten Infected Plants

The various parameters are as follows: *r*_1_, recombination rate between markers a and b; *r*_2_, recombination rate between markers b and c; *r*_3_, recombination rate between markers c and d; *i*_12_, interference between crossovers in segments a–b and b–c; *i*_23_, interference between crossovers in segments b–c and c–d; *i*_13_, interference between crossovers in segments a–b and c–d; *i*_123_, second-order interference accounting for residual interference. The recombination rates are the maximum likelihood estimates (± 95% confidence intervals). The interference parameters were obtained numerically as explained in the Materials and Methods

To relax the assumption of the number of replications during the 21 d, we calculated the recombination parameters assuming five or 20 generations. The effect of the number of generations on the estimates is linear: doubling the number of generations results in a halving of the recombination rate (detailed results not shown). For example, the average recombination rates *r*_1_, *r*_2_, and *r*_3_ assuming 20 generations were equal to 0.05, 0.04, and 0.025, respectively (compare with values in Table 2), yielding per nucleotide per generation recombination rates of 1.9 × 10^−5^, 2.2 × 10^−5^ and 1.6 × 10^−5^.

Inspection of Table 2 also shows that first-order interference coefficients were in general negative, indicating that a crossing over in one genomic segment increases the probability that a crossing over will occur in another genomic segment, while the second-order coefficient parameter had an average value close to zero with a large variance. The mechanism leading to these results will be discussed in the following section.

## Discussion

One major breakthrough in the work presented here lies in the space and time scales at which the experiments were performed. Indeed, the processes occurring within the course of a single infection of one multi-cellular host are of obvious biological relevance for any disease. Previous studies on viral recombination suffered from major drawbacks in this respect, basing their conclusions on experiments relying on complementation among non-infectious viruses or between viruses with undetermined relative fitness, on phylogenetically based analyses, or on experiments in cell cultures. For reasons detailed in the Introduction, the first two methods either do not provide information on the frequency of recombination, but only its occurrence, or address the question at a different temporal, and often spatial, scale. Results from cell cultures, on the other hand, impose cell co-infection by different viral variants, potentially overestimating the frequency of recombination events. Our study circumvents these limitations by analyzing viral genotypes sampled from infected plants after the course of a single infection, and therefore the invasion and co-infection of cells in various organs and tissues is very close to natural.

More than half of the genomes (53.8% ± 2.0%; see Table 1) present in a CaMV population after a single passage in its host plant were identified as recombinants, and these data allowed us to infer a per nucleotide per generation recombination rate on the order of 2 × 10^−5^ to 4 × 10^−5^. The time length of one generation, i.e., the time required for a given genome to go from one replication to the next, is totally unknown in plant viruses. The only experimental data available on CaMV are based on the kinetics of gene expression in infected protoplasts, where the capsid protein is produced between 48 and 72 h [40]. The reverse transcription and the encapsidation of genomic DNA being two coupled phenomena [30], we judged it reasonable to assume a generation time of 2 d and, thus, an average of ten generations during our experiments. In case this estimate is mistaken, we have verified a linear relationship between *r* and the number of generations, thereby allowing an immediate adjustment of *r* if the CaMV generation time is more precisely established. At this point, we must consider that all cloned genomes may not have been through all the successive replication events potentially allowed by the timing of our experiments. It was previously shown that about 95% of CaMV mature virus particles accumulate in compact inclusion bodies [41], where they may be sequestered for a long time, as such inclusions are very frequent in all infected cells, including those in leaves that have been invaded by the virus population for several weeks. The viral population may thus present an age structure that could bias the estimation of the recombination rate. In order to minimize this bias, the clones we analyzed were collected in one young newly formed leaf, where the chances of finding genomes from “unsequestered lines” were assumed to be higher. In any case, our data analysis is conservative, since this age structure can only lead to an underestimation of the recombination rate.

Our results show that interferences between pairs of loci are negative: a recombination event between two loci apparently increases the probability of recombination between another pair of loci. We believe that the most parsimonious explanation of these negative interferences is based on the way the infection builds up within plant hosts. Indeed, one can divide infected host cells into those infected by a single virus genotype and those infected by more than one viral genotype. In the former, analogous to clonal propagation, recombination is undetectable. In the latter, recombination is not only detectable but, as our results indicate, very frequent. Samples consisting of viruses resulting from a mixture of these two types of host cell infections will thus contain viruses with no recombination and viruses with several recombination events, thus yielding an impression of negative interference. These conceptual arguments are supported by mathematical models. It is indeed easy to show (detailed results not shown) that if a proportion *F* of the population reproduces clonally, analogous to single infections, while the remaining reproduces panmictically, negative interferences could be inferred even if they do not exist. For example, assuming a three-locus model with real recombination rates *r*_1_ and *r*_2_ and interference *i*_12_, the “apparent” recombination and interference parameters, would be *r*_1_ = (1 − *F*)r_1_, *r*_2_ = (1 − *F*)*r*_2_, and *i*_12_ = −(*F* − *i*_12_)/(1 − *F*). Interestingly, this example also shows that our estimates of the recombination rate are conservative: that a fraction *F* of host cells are singly infected while others are multiply infected leads to an underestimation of the recombination rate.

As judged by *r*_1_, *r*_2_, and *r*_3_, calculated between markers a–b, b–c, and c–d, respectively, we found evidence for recombination through the entire CaMV genome. The values for *r*_1,_ *r*_2_, and *r*_3_ are remarkably similar, hence the recombination sites seem to be evenly distributed along the genome. We considered the template-switching model as the major way recombinants are created in CaMV. As already mentioned in the Introduction, hot spots of template switching have been predicted at the position of the 5′ extremities of the 35S and 19S RNAs [21,36,42]. If other recombination mechanisms, such as that associated with second-strand DNA synthesis or with the host cell DNA repair machinery, act significantly, hot spots would be expected at the positions of the sequence interruption Δ1, Δ2, and Δ3 [43]. Due to the design of our experiment and the position of the four markers, we have no information on putative hot spots at positions corresponding to the 5′ end of the 35S RNA and to Δ1 (at nucleotide position 0). Nevertheless, the putative hot spots at the 5′ end of the 19S RNA and at Δ2 and Δ3 (nucleotide positions 4,220 and 1,635, respectively) fall between marker pairs c–d, b–c, and a–b, respectively. Our results indicate that either these hot spots are quantitatively equivalent—though predicted by different recombination mechanisms—or, more likely, that they simply do not exist. Whatever the explanation, what we observe is that the CaMV can exchange any portion of its genome, and thus any gene thereof, with an astonishingly high frequency during the course of a single host infection.

To our knowledge, the viral recombination rate has never previously been quantified experimentally for a plant virus [3]. In contrast, retroviruses and particularly HIV-1 have been extensively investigated in that sense. As we have already discussed for these latter cases, the quantification of the intrinsic recombination rate was carried out in artificially co-infected cell cultures. The estimated intrinsic per nucleotide per generation recombination rate in HIV-1 is on the order of 10^−4^ [14,15,19], less than one order of magnitude higher than our estimation for CaMV. Because for various reasons detailed above we probably underestimate the within-host CaMV recombination rate, we believe that the intrinsic recombination rate in CaMV is higher and perhaps on the order of that of HIV.

Other pararetroviruses such as plant badnaviruses or vertebrate hepadnaviruses have a similar cycle within their host cells, including steps of nuclear minichromosome, genomic size RNA synthesis, and reverse transcription and encapsidation. Nevertheless, vertebrate hepadnaviruses (e.g., hepatitis B virus) infect hosts that are very different from plants in their biology and physiology, and this could lead to a totally different frequency of cell co-infection during the development of the virus populations. Thus, even though our results can be informative for other pararetroviruses because of the viruses' shared biological characteristics, they should not be extrapolated to vertebrate pararetroviruses without caution.

## Materials and Methods

### 

#### Viral isolates

We used the plasmid pCa37, which is the complete genome of the CaMV isolate Cabb-S, cloned into the pBR322 plasmid at the unique SalI restriction site [44]. To analyze recombination in different regions of the genome, we introduced four genetic markers: a, b, c, and d, at the positions 881, 3,539, 5,365, and 6,943, respectively, thus approximately at four cardinal points of the CaMV circular double-stranded DNA of 8,024 bp (Figure 1). All markers, each corresponding to a single nucleotide change, were introduced by PCR-directed mutagenesis in pCa37, and resulted in the duplication of previously unique restriction sites BsiWI, PstI, MluI, and SacI in a plasmid designated pMark-S. Because, in this study, we targeted the possible exchange of genes between viral genomes, all markers a, b, c, and d were introduced within coding regions corresponding to open reading frames I, IV, V, and VI, respectively. Another important concern was to quantify recombination in the absence of selection, i.e., to create neutral markers. Consequently all markers consist of synonymous mutations (see below).

#### Production of viral particles and co-inoculation

To generate the parental virus particles, plasmids pCa37 and pMark-S were mechanically inoculated into individual plants as previously described [33]. All plants were turnips (B. rapa cv, “Just Right”) grown under glasshouse conditions at 23 °C with a 16/8 (light/dark) photoperiod. Thirty days post-inoculation, all symptomatic leaves were harvested and viral particles were purified as described earlier [45].

The resulting preparations of parental viruses, designated Cabb-S and Mark-S, were quantified by spectrometry using the formula described by Hull et al. [46]. We fixed the initial frequency of markers to a value of 0.5, and a solution containing 0.1 mg/ml of virus particles of both Cabb-S and Mark-S at a 1:1 ratio was prepared. Plantlets were co-infected by mechanical inoculation of two to three leaves with 20 μl of this virus solution, using abrasive Celite AFA (Fluka, Ronkonkoma, New York, United States). The mixed CaMV population was allowed to grow during 21 d of systemic infection.

#### Estimation of marker frequency within mixed virus populations

We designed an experimental protocol for quantifying marker frequency within a mixed Cabb-S/Mark-S virus population after a single passage in a host plant. Twenty-four individual plants, inoculated as above with equal amounts of Cabb-S and Mark-S, were harvested 21 d post-inoculation, when symptoms were fully developed. The viral DNA was purified from 200 mg of young newly formed infected leaves according to the protocol described previously [47]. After the precipitation step of this protocol, the viral DNA was resuspended and further purified with the Wizard DNA clean-up kit (Promega, Fitchburg, Wisconsin, United States) in TE 1X (100 mM Tris-HCl and 10 mM EDTA [pH 8]). Aliquots of viral DNA preparations were digested by restriction enzymes corresponding either to marker a, b, c, or d and submitted to a 1% agarose gel electrophoresis, colored by ethydium bromide and exposed to UV. Each individual restriction enzyme cut once in Cabb-S DNA and twice in Mark-S, thus generating DNA fragments of different sizes attributable to one or the other in the mixed population of CaMV genomes. After scanning the agarose gels, we estimated the relative frequency of the two genotypes in each viral DNA preparation and at each marker position, by densitometry using the NIH 1.62 Image program. The statistical analyses of the frequency of the four markers are described below.

#### Isolation of individual CaMV genomes and identification of recombinants

To identify and quantify the recombinants within the CaMV mixed populations, aliquots from ten of the 24 viral DNA preparations described above were digested by the restriction enzyme SalI, and directly cloned into pUC19 at the corresponding site. In each of the ten viral populations analyzed, 50 full-genome-length clones were digested separately by BsiWI, PstI, MluI, and SacI, to test for the presence of marker a, b, c, and d, respectively. In this experiment, with the marker representing an additional restriction site, we could easily distinguish between the Cabb-S and the Mark-S genotype at all four marker positions, upon agarose gel (1%) electrophoresis of the digested clones. Clones with none or all four markers were parental genotypes, whereas clones harboring 1, 2, or 3 markers were clearly recombinants. Due to the very high number of recombinants detected, markers eventually appearing or disappearing due to spontaneous mutations were neglected.

#### Statistical analysis

Here we present the different methods we used to quantify recombination in the CaMV genome. Because all these methods assume that the different markers are neutral, we first discuss assumption.

We used two datasets to test the neutrality of markers, both resulting from plants co-infected with a 1:1 ratio of Mark-S and Cabb-S. The first consisted of viral DNA densitometry data derived from 24 plants (described above), where for each plant we have an estimate of the frequency of each marker in the genome population. The second consisted of the restriction of 50 individual full-genome-length viral clones obtained from one co-infected plant (described above), yielding an estimate of the frequency of each marker, and this was repeated on ten different plants. The frequencies of the different markers were 0.508, 0.501, 0.516, and 0.507 for markers a, b, c, and d in the first dataset and 0.521, 0.518, 0.514, and 0.524 in the second dataset. We tested whether these frequencies were significantly different from the expected value under neutrality, 0.5, using either *t*-tests, for datasets where normality could not be rejected (seven out of eight cases), or Wilcoxon signed-rank non-parametric tests otherwise (marker c in the first dataset). In all cases *p*-values were larger than 0.05.

There are several cautionary remarks regarding these analyses. First, in all cases we found an excess of markers. Unfortunately, the two datasets cannot be regarded as independent because, even though the methods through which the frequency estimates were obtained were different, the plants used in the second dataset were a subset of the plants of the first. We thus have only four independent estimates in each case, and there is minimal power to detect significant deviations from neutrality with such a small sample size. It should be noted at this stage that deviations from the expected value could also be caused either by slight deviations from the 1:1 ratio in the infecting mixed solution, or by deviations from that ratio in the frequency of the viral particles that actually get into the plants. Second, because of the relatively small sample sizes and low statistical power, the tests presented above could have detected only large deviations.

The results clearly show, however, precisely that the markers do not have large effects, if any, and that therefore recombination estimates would be affected only very slightly by any hypothetical selective effects of the markers. Because of this, along with the fact that the introduced markers provoke silent substitutions in the CaMV genome, we assumed that markers were effectively neutral in the rest of the analysis.

The dataset used to estimate the recombination frequency consisted of the 500 full-genome-length viral clones (50 from each of ten co-infected plants) individually genotyped for each of the four markers. As discussed in detail in the Results, recombination was very frequent and concerned all four markers. Indeed, approximately half of the genotyped clones exhibited a recombinant genotype. It was therefore meaningful to try to obtain quantitative estimates of recombination from our data.

Our aim was to analyze viral recombination in a live host. Consequently, we had to deal with the fact that more than one viral replication cycle occurred during the 21 d that infection lasted in our experiment (we had to wait that long for the disease to develop and to be able to recover sufficient amounts of viral DNA from each infection). Based on the kinetics of gene expression [40], we postulate that each replication cycle lasts between 2 and 3 d, and that therefore seven to ten cycles occurred between infection and the sampling time. In case this assumption is incorrect, we did calculations assuming five, seven, ten, or 20 replication cycles during these 21 d. As shown, the results were not affected qualitatively, and only slightly quantitatively. It is important to note that we assumed that recombination occurred through a template-switching mechanism, and that therefore, from a recombination point of view, the CaMV genome is linear. The reverse transcription starts and finishes at the position 0 in Figure 1, which is the point of circularization of the DNA genome. This implies that changes between contiguous markers a–b, b–c, and c–d can be considered as true recombination whereas those between a and d cannot, as they may simply stem from circularization of DNA, during the synthesis of which the polymerase has switched template once anywhere between a–b, b–c, or c–d.

To estimate the recombination rate between markers, we wrote recurrence equations describing the change in frequency of each genotype over one generation, assuming random mating and no selection (i.e., the standard Wright–Fisher population genetics model). We then expressed the frequency of all possible genotypes *n* generations later as a function of their initial frequency and of the recombination parameters. Subsequently we calculated the maximum likelihood estimates of the recombination parameters and their asymptotic variances given initial frequencies (we assumed that the two “parental” genotypes, Mark-S and Cabb-S, had equal initial frequencies of 0.5 and that all other genotypes had initial frequencies of zero) and frequencies after *n* generations (the observed frequencies; as stated above we used different values of *n*). All algebraic and numerical calculations were carried out with the software Mathematica.

The recombination parameters are the recombination rates between two adjacent loci, e.g., *r*_1_ for the recombination rate between markers a and b, and the interference coefficients, e.g., *i*_12_ for interference between recombination events in the segments between markers a and b and b and c. To define these parameters we followed Christiansen [48], and in particular the recombination distributions for two, three, and four loci (respectively, Tables 2.7, 2.8, and 2.9 of [48]). It is important to realize that given the definitions of these parameters, the estimator of the recombination rate between two loci is not affected by the number of loci considered. In other words, we obtain the same estimation of the recombination rate between markers a and b whether we consider genotypic frequencies at just these two loci, or the frequencies at these two loci plus a third locus, or the complete information to which we have access, the four-marker genotypes. Information on additional loci only affects the estimates of the interference coefficients.

It proved impossible to carry out the calculations for four loci algebraically. Instead, we used a computer program to calculate the expected genotypic frequencies at all four loci after *n* generations, given the above stated initial frequencies and specified recombination parameters. For each combination of recombination parameters we calculated a Euclidean distance between the vector of the expected genotypic frequencies and the observed genotypic frequencies, and considered that the estimated recombination parameters were those yielding the minimal Euclidean distance. In all cases, the estimated recombination rates between pairs of loci were equal to the second decimal to those estimated algebraically from data for three or two loci.

## Abbreviation

CaMV: cauliflower mosaic virus
